# Cubic Microcontainers Improve In Situ Colonic Mucoadhesion and Absorption of Amoxicillin in Rats

**DOI:** 10.3390/pharmaceutics12040355

**Published:** 2020-04-14

**Authors:** Juliane Fjelrad Christfort, Antonio José Guillot, Ana Melero, Lasse Højlund Eklund Thamdrup, Teresa M. Garrigues, Anja Boisen, Kinga Zór, Line Hagner Nielsen

**Affiliations:** 1 Department of Health Technology, Technical University of Denmark, Ørsteds Plads, 2800 Kgs. Lyngby, Denmark; lhth@dtu.dk (L.H.E.T.); aboi@dtu.dk (A.B.); kinzo@dtu.dk (K.Z.); lihan@dtu.dk (L.H.N.); 2 Department de Farmàcia I Tecnología Farmacèutica, Avda. Vincent Andrés Estellés s/n, 46100 Burjassot (Valencia), Spain; antonio.guillot@uv.es (A.J.G.); teresa.garrigues@uv.es (T.M.G.)

**Keywords:** in situ perfusion, microdevices, shape, mucoadhesion, colon absorption

## Abstract

An increased interest in colonic drug delivery has led to a higher focus on the design of delivery devices targeting this part of the gastrointestinal tract. Microcontainers have previously facilitated an increase in oral bioavailability of drugs. The surface texture and shape of microcontainers have proven to influence the mucoadhesion ex vivo. In the present work, these findings were further investigated using an in situ closed-loop perfusion technique in the rat colon, which allowed for simultaneous evaluation of mucoadhesion of the microcontainers as well as drug absorption. Cylindrical, triangular and cubic microcontainers, with the same exterior surface area, were evaluated based on in vitro release, in situ mucoadhesion and in situ absorption of amoxicillin. Additionally, the mucoadhesion of empty cylindrical microcontainers with and without pillars on the top surface was investigated. From the microscopy analysis of the colon sections after the in situ study, it was evident that a significantly higher percentage of cubic microcontainers than cylindrical microcontainers adhered to the intestinal mucus. Furthermore, the absorption rate constants and blood samples indicated that amoxicillin in cubic microcontainers was absorbed more readily than when cylindrical or triangular microcontainers were dosed. This could be due to a higher degree of mucoadhesion for these particular microcontainers.

## 1. Introduction

Oral drug delivery remains the preferred administration route due to ease of use, flexibility and patient compliance [1]. Despite many advances in oral delivery systems [2,3,4], the design of ‘the ideal delivery device’ is still widely discussed and depends on the application.

In the past decades, an increased interest in colon drug delivery has led to significant research with a focus on designing delivery devices that target this part of the gastrointestinal (GI) tract [5]. In addition to local delivery, the colon has also been suggested as an interesting site for systemic drug delivery due to increased oral bioavailability for some drugs and longer transit time compared to the small intestine [6,7,8]. The prolonged transit time allows timing of the treatment to periods with maximal disease activity; for example, in diseases where symptoms are more pronounced in the morning (hypertension, asthma and arthritis) [7].

Mucoadhesion is an important factor in relation to targeted delivery to any part of the GI tract, since it can prolong the residence time and facilitate drug release in close proximity to the epithelium. To understand mucoadhesion, six general theories have been proposed [9]. Amongst these are the wetting theory and the mechanical theory. The wetting theory is mainly applied to liquid or low-viscosity systems [9], while the mechanical theory can be applied to more rigid and adhesive materials. The mechanical theory explains mucoadhesion in terms of interlocking into irregularities on a rough surface [9,10]. Due to the complex nature of mucoadhesion, it is not likely that the phenomenon can be described by one of these theories alone [9]. Properties affecting mucoadhesion have been thoroughly investigated and it has been indicated that size and shape have a high impact on the mucoadhesive strength for micro- and nano-scale particles [11,12,13]. Advanced polymeric particles have paved the way for many new controlled drug delivery strategies [14]. However, as the field of drug delivery is moving towards more advanced microdevices, additional knowledge is needed to fully disclose and understand the influence of the multitude of parameters influencing mucoadhesion of drug delivery devices and the associated absorption of drugs.

Unidirectionally-releasing microdevices have been proposed for oral drug delivery to ensure a high local concentration of the active pharmaceutical ingredient (API) at the absorption site and to prolong the residence time [15,16]. The prolonged residence time is proposed to occur by decreased shear stress and increased retention [17]. For example, planar microdevices with a diameter of 200 µm were shown to enhance in vivo retention after oral dosing in mice [15]. These microdevices were found to adhere better than microspheres with the same surface area in the proximal and medial intestine. In the colon, however, the microspheres were retained approximately two times more than the microdevices [15]. The concept of planar microdevices was further developed with the inclusion of nanostraw structures on the surface, which was shown to enhance bioadhesion in a Caco-2 cell flow system when compared to similar microdevices without nanostraws [18]. Microcontainers, one type of unidirectionally-releasing microdevice, have previously been shown to improve oral bioavailability of drugs and provide inherent mucoadhesion [16,19]. Microcontainers are micrometer-sized devices (fabricated in polymers) with an inner cavity for storage of the API. Coating of the API-loaded cavity protects the content from the harsh environment of the stomach and provides unidirectional release at a relevant site in the GI tract [16]. Due to versatile fabrication processes, microcontainers have been fabricated in different materials, shapes and size ranges [20,21,22].

The influence of material composition, shape and size on the interaction with mucus has also been evaluated for microcontainers in an intestinal ex vivo perfusion model [22,23,24]. Here, triangular microcontainers adhered more readily in the mid-part of a porcine small intestinal section than cylindrical ones. Similarly, larger cone shaped microcontainers generally adhered better than cylindrical microcontainers with the same size [22,24]. When evaluating these microdevices fabricated in different materials in an ex vivo perfusion model, poly(lactic-*co*-glycolic acid) (PLGA) (50:50) microcontainers showed slightly higher mucoadhesion compared to polycaprolactone (PCL) and PLGA (75:25) microcontainers [23]. In a different study performed in the same model, there was a tendency that SU-8 microcontainers adhered better to the mucosa than PCL microcontainers with the same dimensions [22].

Techniques like the perfusion model described above, or atomic force microscopy (AFM), are commonly applied to study mucoadhesion ex vivo [25,26]. However, these techniques do not allow for simultaneous evaluation of the permeation across the intestinal barrier. In order to study API permeation, methods like in vitro studies with Caco-2 cells or ex vivo studies using an Ussing chamber setup are traditionally used [27,28].

A well-documented method to explore the performance of drug delivery systems in situ is the intestinal perfusion technique in rodents, introduced by Schanker in 1958 [29]. Several decades later, the technique is still widely applied in the field of intestinal absorption research due to its versatility [30,31,32]. Furthermore, the in situ technique provides the opportunity to investigate absorption and mucoadhesion simultaneously in a specific region of the intestine. These are important characteristics for evaluation of both local delivery and formulations aiming for prolonged release followed by absorption.

This in situ perfusion technique is applied in anesthetized rodents, where the relevant part of the intestine is isolated from nearby tissues, but not removed from the living organism. The perfusion can be performed as single-pass intestinal perfusion or with a closed-loop technique [32]. In both cases, the neural, endocrine, blood and lymphatic contributions are maintained during the experiment in order to simulate in vivo conditions. Both perfusion techniques have shown equally good correlations to literature values of the absorbed fraction of the oral dose (F_abs_) in humans [32,33]. The closed-loop in situ perfusion technique based on Doluisio’s method [34] has been widely applied for intestinal absorption of different drugs and in different regions of the intestine [35,36,37]. Earlier, the closed-loop intestinal perfusion technique has been applied to investigate the mechanisms governing absorption from drug suspensions [38] and drug-loaded microdevices [16] in the small intestine. The closed-loop perfusion technique has previously been validated to study colonic absorption [37], but has not yet been applied to study mucoadhesion and absorption simultaneously in this region of the GI tract.

Previously, we have seen that the shape and surface texture of microdevices influence mucoadhesion ex vivo [22,39]. More specifically, triangular microcontainers previously resulted in improved mucoadhesion in parts of a small intestinal section compared to cylindrical microcontainers [22]. However, these microcontainers were not normalized with respect to the surface area. Thus, it is unclear whether the shape or the difference in surface area was the reason for this observed effect. To elaborate on this, the present study aimed to evaluate differently shaped microcontainers, with the same exterior surface area, using a closed-loop in situ intestinal perfusion technique. This technique allowed us to simultaneously evaluate mucoadhesion of microcontainers and absorption of a model drug, amoxicillin, in the colon of rats. Cylindrical, triangular and cubic microcontainers were evaluated regarding in vitro release, in situ mucoadhesion and in situ absorption of amoxicillin. Additionally, the mucoadhesion of empty cylindrical microcontainers with pillars on the top surface were compared to a control without pillars.

## 2. Materials and Methods

### 2.1. Materials

Amoxicillin trihydrate was bought from TCI (Tokyo, Japan) and Eudragit^®^ L100 was acquired from Evonik Industries (Essen, Germany). Dibutyl sebacate, isopropanol and potassium phosphate monobasic for the HPLC mobile phase were purchased from Sigma Aldrich (St. Louis, MO, USA). Methanol was bought from VWR International (Radnor, PA, USA).

The negative epoxy based photoresist SU-8 was used for production of microcontainers. Formulations with two different viscosities were used (i.e., SU-8 2035 and 2075) and the cross-linked structures were developed in Developer mr-Dev 600. Resist and developer were purchased from Micro Resist Technology GmbH (Berlin, Germany). Single-side polished ø100 mm Si substrates with a thickness of 525 µm were acquired from Topsil Globalwafers A/S (Frederikssund, Denmark).

For the phosphate buffered saline (PBS) used in the in situ perfusion studies, sodium chloride, potassium chloride, sodium phosphate dibasic and potassium phosphate monobasic were purchased from Scharlab (Barcelona, Spain). Animals were from Charles River Laboratories (Quebec, QC, Canada). Sylgaard 184 silicone elastomer kit was purchased from Dow Chemical (Midland, MI, USA). Ultrapure water used throughout the studies was obtained from a Q-POD^®^ dispenser (Merck Millipore, Burlington, MA, USA).

### 2.2. Fabrication of SU-8 Microcontainers

Microcontainers with five different designs were used in these experiments. Amoxicillin-loaded microcontainers enteric coated with Eudragit^®^ L100, were produced as 3D structures in three different shapes; cylindrical, equilateral triangular prism and quadrangular prism (Table 1). The central cylindrical compartment for drug loading was designed with a constant volume for all shapes. Furthermore, the three shapes were designed to have a constant outer surface area when neglecting the top surface (which was coated). By maintaining a constant interaction area, the studies aim to isolate the influence of geometry on the colonic mucoadhesion. In addition to the above mentioned microcontainers, cylindrical microcontainers with and without 35 µm pillars (in diameter) on the sidewalls were produced (Table 1).

All microcontainers were produced following an approach first introduced for drug delivery devices in [40], then adapted and modified in [41]. Starting out with clean Si substrates, a release layer consisting of 5 nm Ti and 20 nm Au was deposited (Temescal FC-2000, Ferrotec Corporation, Santa Clara, CA, USA) using electron-beam evaporation. The release layer ensures adequate adhesion during drug loading and lid coating, but allowed for harvesting the microcontainers without damaging the SU-8 structures. All microcontainers featured an approximately 35 µm thick bottom layer formed by spin coating SU-8 2035 (RCD8 manual spin coater, Süss MicroTec, Garching, Germany). This was followed by performing a soft bake at 50 °C for 2 h (ramping rate 2 °C/min, used for all baking steps), conducting UV exposure using doses in excess of 200 mJ/cm^2^ and then carrying out a post exposure bake (PEB) at 50 °C for 6 h using the aforementioned ramping rate. The UV exposure was conducted using a maskless aligner (MLA100 Tabletop Maskless Aligner, Heidelberg Instruments, Heidelberg, Germany) or a conventional mask aligner (Karl Süss Mask Aligner MA6, Süss MicroTec, Garching, Germany) operated in soft contact mode. The sidewalls of the microcontainers were defined in SU-8 2075, which was spin coated and subject to a soft bake at 50 °C for 10 h. The UV exposure was conducted using a dose of 500 mJ/cm^2^. For defining the cylindrical empty reference microcontainers with no pillars on the sidewalls, the mask aligner was operated in proximity exposure mode to avoid direct contact between the soda-lime glass mask and the SU-8. The remaining four microcontainer types were UV exposed using the maskless aligner. After UV exposure, a PEB of 10 h at 50 °C was carried out. To form the pillars on the sidewalls of one of the empty control microcontainer groups, a final layer of SU-8 2035 was spin coated, soft baked at 50 °C for 2 h, UV exposed using a dose of 200 mJ/cm^2^ and finally subjected to a PEB at 50 °C for 6 h. The substrates, carrying the multitude of different drug delivery devices, were developed by immersion in two separate baths for 2 × 20 min and flushed with copious amounts of isopropanol before leaving the substrates to dry. Prior to drug loading and lid coating, the Si substrates were diced (Automatic Dicing Saw DAD 321, DISCO, Tokyo, Japan) into 12.8 × 12.8 mm^2^ chips, each containing 625 microcontainers arranged in a 25 × 25 array.

The microcontainers were characterized using both conventional bright-field microscopy (Nikon Eclipse L200, Nikon Metrology, Tokyo, Japan) for extracting data on the horizontal dimensions and vertical scanning interferometry (PLu Neox 3D Optical Profiler, Sensofar Metrology, Barcelona, Spain) aimed at characterizing the vertical dimensions (i.e., inner and outer heights and pillar height). The results of the topology characterization are summarized in Table 1. For the horizontal measurements (i.e., diameters and side lengths), a value corresponding to the optical resolution multiplied by a factor of 3 was used. The optical resolution, R, of the used objective (20×, NA = 0.45) was evaluated as R = (0.61λ)/NA where the illumination wavelength was λ = 550 nm. This resulted in the stated measurement uncertainty of ±2.2 µm. When considering the height measurements, we used the standard deviation based on an ensemble of measurements. Generally the horizontal dimensions are subject to small variations and the spread in the vertical dimensions are governed by the homogeneity of the spin coated SU-8 layers.

### 2.3. Loading of Amoxicillin into Microcontainers and Spray Coating with Eudragit^®^ L100

For the in situ perfusion study, the microcontainer chips were loaded with amoxicillin as previously described [16,42]. Briefly, amoxicillin was manually distributed on the microcontainer chip and excess drug between the microcontainers was subsequently removed with an air gun. For the in vitro release studies, the microcontainers were loaded using a PDMS shadow mask to cover the chip area between the microcontainers, as previously described [43]. In both cases, the chip with microcontainers was weighed before and after loading to determine the loaded amount of amoxicillin. Visualization of the loading was carried out by scanning electron microscopy (SEM) using a Hitachi TM3030Plus tabletop microscope (Hitachi High-Technologies Europe, Krefeld, Germany).

A lid of Eudragit^®^ L100 was deposited over the microcontainers as previously described [19], by spray coating an isopropanol solution with 1% *w/v* Eudragit^®^ L100 and 5% *w/w* (in relation to the polymer) dibutyl sebacate. Briefly, the solution was sprayed over the amoxicillin-loaded microcontainers one chip at a time using an ultrasonic spray coater (Exactacoat system, Sono-Tek, Milton, NY, USA) with an Accumist nozzle operating at 120 kHz. Each chip was coated with 30 loops of two alternating spray paths having an offset of 2 mm, resulting in a total of 60 passages. Visualization of the coated microcontainers was carried out by SEM.

### 2.4. In Vitro Release Studies

The release of amoxicillin from the differently shaped microcontainers coated with Eudragit^®^ L100 was measured using a µDISS Profiler^TM^ (Pion Inc., Billerica, MA, USA), as previously described in the literature [16,19,42]. Initially, a calibration curve was prepared by addition of different volumes of amoxicillin in PBS stock solution to 10 mL PBS (pH 7.4) followed by measurements of UV absorbance in the range of 270–280 nm. For the release study, a microcontainer chip was placed on top of a cylindrical magnetic stirring bar with double-sided carbon tape, transferred to a sample vial and covered with 10 mL PBS at the same time as the experiment was started. Studies were performed at 37 °C with a stirring rate of 100 rpm, and the path length of the UV probes was 5 mm. UV measurements were carried out every 10 s until an amoxicillin release of 100% was observed after approximately 60 min.

### 2.5. Closed-Loop Colon Perfusion Study in Rats

Male Wistar rats were used in accordance with 2010/63/EU directive of 22 September 2010 regarding the protection of animals used for scientific experimentation. The Ethics Committee for Animal Experimentation of the University of Valencia approved the experimental protocols (code A1544541996825). Male Wistar rats weighing 240 ± 12 g were fasted for 3 h before the experiments with ad libitum access to water. The rats were anesthetized by intraperitoneal injection of pentobarbital sodium (40 mg/kg) prior to the surgical procedure.

**Figure 1:**
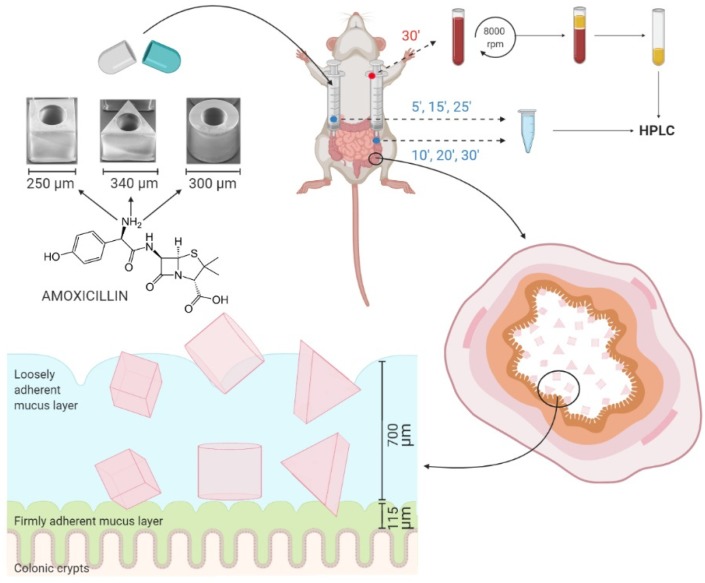
Schematic overview of the in situ colonic perfusion study. The different microcontainers were dosed through two cannulas connected to glass syringes creating a closed colon compartment. To investigate the absorption of amoxicillin, intestinal samples were collected from the two glass syringes every 5 min throughout the experiment and a blood sample was collected 30 min after the experiment. The plasma and intestinal samples were analyzed with high performance liquid chromatography (HPLC) to determine the concentration of amoxicillin. The mucoadhesion of the microcontainers was evaluated as the percentage adhering to the colonic section after the study.

The drug absorption rate constant of amoxicillin in the colon and the mucoadhesion of microcontainers were evaluated by the in situ closed-loop perfusion method based on Doluisio’s technique [34]. Briefly, the animals were placed under a heating lamp and a midline abdominal incision was made to expose the intestine. The colon section was isolated by making two incisions; one after the caecum and the other just before the rectum. Two glass syringes connected to three-way stopcock valves were introduced in the incisions with the help of two cannulas, creating an isolated compartment as depicted in Figure 1. Procedures were performed with care to avoid disturbance of the intestinal blood supply and intestinal bleeding. In order to remove all intestinal content and wash the colon, the intestinal section was thoroughly flushed with PBS pre-heated to 37 °C. The colon was carefully placed back into the peritoneal cavity and the abdomen was covered with cotton wool pads to prevent peritoneal liquid evaporation and heat loss [16,32,35].

A number of microcontainers, corresponding to 0.4 mg amoxicillin (120 cylindrical, 278 cubic or 136 triangular microcontainers) were dispersed in 5 mL pre-heated PBS and introduced through the cannulas into the isolated section. For the empty reference cylinders without and with pillars, 204 ± 25 and 223 ± 45 microcontainers, respectively, were dosed in the same manner. Samples of 150 µL were collected every 5 min up to a period of 30 min (Figure 1). Sample withdrawal was performed by pushing the luminal content from one syringe to the other, alternatively from the proximal syringe to the distal one and the other way around [44]. Intestinal samples were stored at −20 °C until further analysis.

Water flux absorption processed during the experiment could be significant, and hence it must be considered [45]. To do so, the volume of the intestinal content was measured in every animal after the whole procedure (V_t_) and compared to the initial volume (V_0_) of 5 mL. The drug concentration in the samples was corrected according to Equation (1):

C_t_ = C_e_ (V_t_/V_0_), (1)

where C_t_ represents the concentration in the absence of water reabsorption at time t, and C_e_ is the experimental value. The corrected concentration, C_t_, was then used to calculate the actual absorption rate coefficient in relation to the initial concentration (C_0_) [45]. The absorption rate coefficients (k_a_) of the compounds were determined by nonlinear regression analysis of the remaining concentrations in the intestinal lumen (C_t_) versus time according to Equation (2):

C_t_ = C_0_ e^−k^_a_^t^ (2)

After 30 min, a cardiac puncture was performed under anesthesia to collect the blood from the rat (Figure 1). The blood was collected in heparinized tubes and centrifuged at 10 °C and 6100× *g* for 8 min. The plasma was stored at −20 °C until further analysis. After the experiment, the isolated colon section was cut and placed onto a glass slide with the luminal side upwards to determine the number of microcontainers. A light microscope (Nikon Eclipse 50i, Nikon, Tokyo, Japan) with camera (Nikon digital camera, DXM1200C, Nikon, Tokyo, Japan) was used to visualize the microcontainers on the colon section.

### 2.6. High Performance Liquid Chromatography Analysis of Intestinal and Plasma Samples

The concentration of amoxicillin in the intestinal fluid and plasma was determined by high performance liquid chromatography (HPLC). HPLC analyses were performed on a Shimadzu HPLC system (Shimadzu, Kyoto, Japan). The system consisted of a CBM-20A system controller, SIL-20AC HT auto sampler, LC-20AD pump, DGU-20A5R degassing unit, CTO-20AC column oven, RID-20A refractive index detector, and SPD-30A photodiode array detector. The mobile phases consisted of A: phosphate buffer (6.8 g KH_2_PO_4_/L, pH 5) and B: acetonitrile, and the samples were run with an isocratic method with an A:B mobile phase ratio of 95:5 v/v. A Luna 5.0 µm C18 100 Å, 250 × 4.6 mm column (Phenomenex ApS, Værløse, Denmark) was used for the analyses and samples were run at 25 °C.

The intestinal samples were vortexed and centrifuged at 10,600× *g* for 10 min and the supernatant was transferred to HPLC vials with 300 µL flat bottom inserts (Frisenette, Knebel, Denmark). For the plasma samples, 60 µL plasma was mixed with 100 µL methanol and otherwise treated as described above for the intestinal samples. Calibration curves were prepared from stock solutions of amoxicillin in water. For the plasma sample analysis, accurate volumes of the stock solutions were mixed with plasma and methanol (same ratio as the samples) and treated the same way as the samples. A volume of 20 µL was injected and the flow rate was 0.8 mL/min with a run time of 10 min for each sample. The absorbance was measured at 230 nm.

### 2.7. Data Analysis

All data processing was performed in Microsoft Excel 2016 (Redmond, WA, USA), GraphPad Prism version 6.0 (GraphPad software, San Diego, CA, USA) and SPSS version 22.0 (IBM Corp, Armonk, NY, USA). The data is expressed as the mean ± standard deviation (SD) unless otherwise stated. Statistical differences were determined using one-way ANOVA followed by Games–Howell post-hoc analysis, where *p*-values below 5% were considered significant.

## 3. Results and Discussion

### 3.1. Microcontainer Characterization and Preparation

For the present study, which addresses the impact of microcontainer geometry on the colonic mucoadhesion and absorption during in situ experiments in rats, three 3D container designs were investigated: cylindrical, cubic and equilateral triangular (Table 1). The microcontainers all had a cylindrical center compartment for drug storage and the outer surface area (neglecting the top surface) was kept constant to ensure an identical interaction area between microcontainers with different shapes and the mucosal layer in the colon.

**Figure 2:**
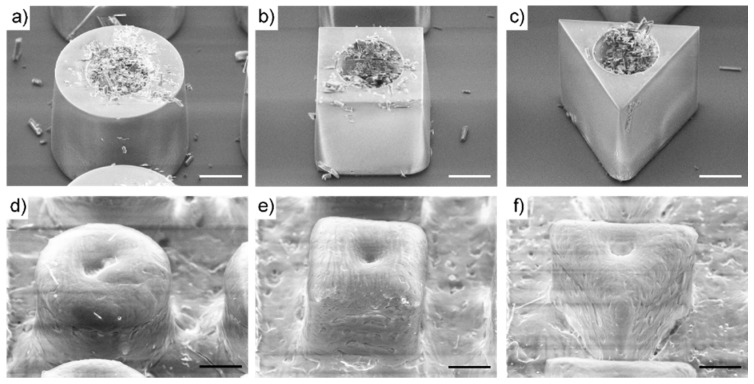
SEM images of a (**a**) cylindrical, (**b**) cubic and (**c**) triangular microcontainer loaded with amoxicillin and a (**d**) cylindrical, (**e**) cubic and (**f**) triangular microcontainer loaded with amoxicillin and coated with Eudragit^®^ L100. All scale bars represent 100 µm.

The microcontainers were loaded with 1.50 ± 0.27 mg, 0.89 ± 0.19 mg and 1.78 ± 0.18 mg amoxicillin per chip for the cylindrical, cubic and triangular microcontainers, respectively (Figure 2a–c). Despite having the same inner cavity volume for drug loading, the cubic microcontainers were loaded with significantly less amoxicillin than the two other shapes. This is ascribed to the manual loading process, where the additional corners can affect the loading efficiency. After drug loading, the microcontainers were coated with Eudragit^®^ L100. Inspection with SEM showed uniform coatings covering the cavities of the drug-loaded microcontainers for all three shapes (Figure 2d–f).

Besides the three shapes described above, cylindrical reference microcontainers with pillars on the top surface of the sidewalls were also fabricated (but not loaded with drug) and the mucoadhesion of these were compared to the mucoadhesion of traditional empty cylinders of similar dimensions as the control (Table 1).

### 3.2. In Vitro Release Studies

**Figure 3:**
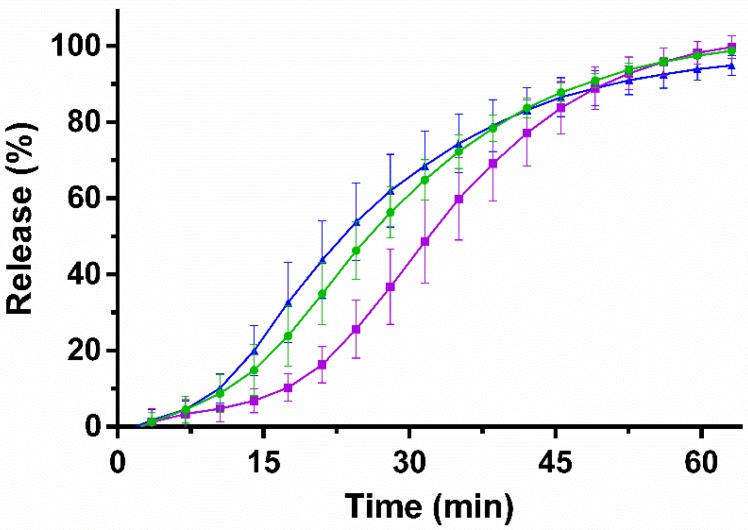
Amoxicillin in vitro release profiles as a function of time from 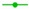 cylindrical, 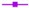 cubic and 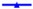 triangular microcontainers in PBS (pH 7.4). All microcontainers were coated with Eudragit^®^ L100. Data represent mean ± SD, *n* = 4.

To investigate the release rate, the in vitro release of amoxicillin from the microcontainers was evaluated on a µDISS Profiler^TM^ in PBS at pH 7.4 (Figure 3).

For all three formulations, the loaded amount of amoxicillin was released after 60 min (98 ± 1%, 98 ± 3% and 94 ± 3% for the cylindrical, cubic and triangular microcontainers, respectively). The observed in vitro release of amoxicillin from the Eudragit^®^ L100-coated microcontainers was expected since the coating dissolves at pH values above 6.0. After 30 min, a release of approximately 60% was observed for the cylindrical and triangular microcontainers, whereas there was a trend towards slower release of amoxicillin from the cubic ones (44 ± 10% after 30 min).

After 45 min, at least 80% of the initial amoxicillin dose was released, which categorizes the formulation as an immediate release formulation according to the European Pharmacopoeia [46]. Comparable pH-dependent release profiles have previously been observed for drugs loaded in microcontainers and coated with Eudragit^®^ L100 [16,47].

### 3.3. In Situ Closed-Loop Colon Perfusion Study in Rats

The in situ closed-loop perfusion technique was applied to study the interaction between microcontainers and the colonic mucus layer, and whether this interaction affected the absorption of amoxicillin from the microcontainers compared to a control solution.

#### 3.3.1. Mucoadhesion of Microcontainers

**Figure 4:**
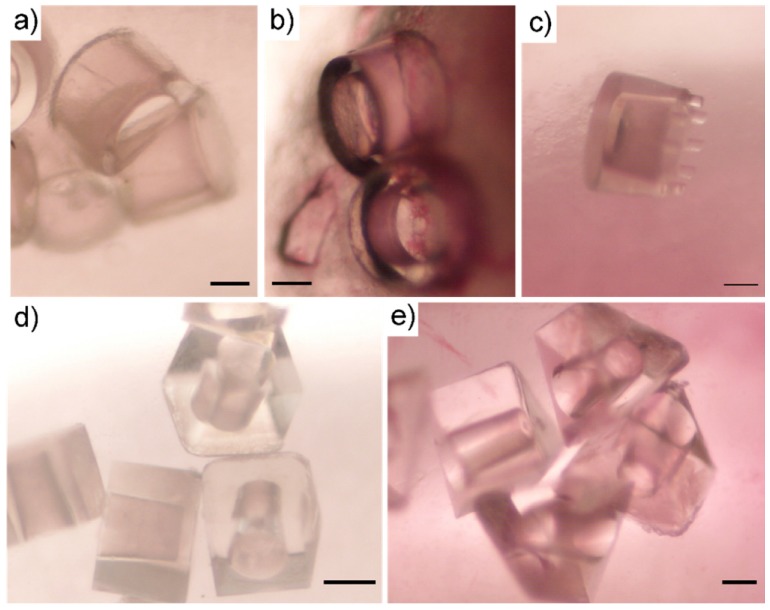
Microscopy images of microcontainers in colonic mucus following in situ perfusion studies. (**a**,**b**) cylindrical microcontainers, (**c**) pillared reference microcontainers, (**d**) cubic and (**e**) triangular microcontainers. All scale bars represent 100 µm.

After 30 min of the perfusion study, the microcontainers were manually localized and counted by inspecting the colon sections under a light microscope (Figure 4). When qualitatively investigating the microcontainers retained in the colonic mucus, it was observed that the microcontainers were mainly found in clusters that were partly or completely covered by mucus (Figure 4). Similar clustering trends have previously been observed for other microdevices evaluated on a cell monolayer under flow [17].

**Figure 5:**
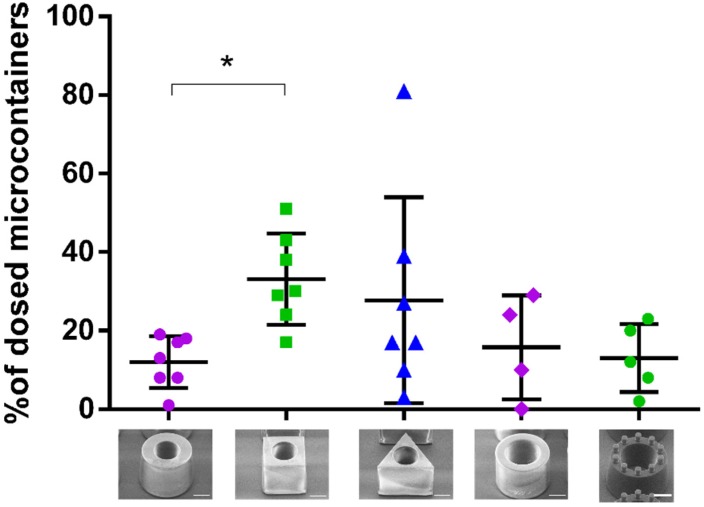
Mucoadhesion of the five microcontainer formulations expressed as percentage of dosed microcontainers still adhering to the mucus after the closed-loop intestinal perfusion study (mean ± SD, *n* = 4–7). * indicates a significant difference with a *p*-value below 5%.

The mucoadhesion of the microcontainers was quantified as the percentage of microcontainers adhering to the colonic mucus after 30 min compared to the total amount of microcontainers dosed (Figure 5). It was found that 12 ± 7% of the loaded cylindrical microcontainers were retained in the colonic mucus. In contrast, a significantly higher (*p* = 0.019) number of cubic microcontainers (33 ± 12%) were found to be retained in the colon sections after the same period of time (Figure 5). The percentage of the triangular microcontainers in the colonic mucus was 28 ± 26% and a higher inter-individual variation was observed for these rats compared to the rats in the other groups (Figure 5).

Based on the data presented in Figure 5, the only significant difference was between the cubic and cylindrical microcontainers loaded with amoxicillin. The absence of significant differences between the other groups can be explained through the rather large data distribution in the group dosed with triangular microcontainers, which varied between 3 and 81% (Figure 5). As expected, the shape with the least pronounced mucoadhesion was the cylindrical one, since this shape did not provide any corners or edges to allow for interaction with the mucus. The most mucoadhesive shape seemed to be the cube, although the differences were non-significant due to the large variations observed for triangular microcontainers. If the cubic and triangular microcontainers are analyzed based on geometry/topology, there are obvious differences between the two shapes. The cubic structures have 6 surfaces with approximately the same area and shape, 12 edges and 8 corners, whereas the triangles have 5 surfaces, 9 edges and 6 corners. We believe that the number of surfaces, corners and edges strongly influence the way the microcontainers are retained in the mucus. These shape differences would also result in different contact surfaces between the microcontainers and the mucus, and thus, differences in mucoadhesion according to the wetting theory [9]. The cylinders will for example have a much smaller contact surface with the mucus if they land on their curved side.

A previous study investigated the mucoadhesion of cylindrical and triangular microcontainers in an ex vivo perfusion model and found triangular microcontainers to be significantly more mucoadhesive in the mid-part of the intestinal section [22]. This finding, in relation to the results in the present study about cubic microcontainers being more adhesive than cylindrical microcontainers, suggest that the presence of corners or edges can influence the mucoadhesion properties. However, the changing properties of the mucosa along the GI tract makes it difficult to directly extrapolate findings from one intestinal region to another.

We did not expect the material SU-8 to have any significant influence on mucoadhesion in itself. Even if SU-8 interacted with the mucus layer, the effect of this interaction would be similar for all the shapes since they have been normalized to have the same exterior surface area. Hence, we would not expect the differently shaped microcontainers to adhere differently as a result of interfacial interactions relating to material properties. Thus, we expect that the observed interaction with the intestinal mucosa is mostly due to mechanical detainment due to the differences in shape rather than chemical interactions.

In addition to the influence of the shape itself, the mucoadhesive effect of pillars applied to the top surface of cylinders was investigated (Figure 5). The amount of reference cylindrical microcontainers with pillars adhering to the colonic mucus was found to be 16 ± 13% after 30 min, which was very similar to the percentage of conventional reference and loaded cylinders (13 ± 9% and 12 ± 7%, respectively). In accordance with the mechanical theory of mucoadhesion described in the introduction, the pillars were introduced on the reference cylinders as bioinspired structures to increase adhesion by filling the imperfections of a rough surface. However, unexpectedly, no differences in mucoadhesion were observed by adding these small structures on top of the reference microcontainers. This could be attributed to the size or number of pillars, which might have been insufficient in order to interact with the mucus in a significant way. Finally, it was observed that handling of the reference microcontainers with pillars resulted in loss of some pillars when the microcontainers were evaluated with SEM before dosing, which could outweigh the potential adhesion effect of the pillars. To fully investigate the concept of surface structures further, additional studies are needed. Nevertheless, the microcontainers themselves seem to present adequate geometrical forms which can be detained in the mucus layer without help from smaller structures on the surface.

Surface modified microdevices have previously been found to increase adhesion in vitro and ex vivo [18]. However, these microdevices were not investigated in situ or in vivo and the surface structures on these devices were remarkably smaller (60–160 nm) than the pillars on the surface of the microcontainers in the present study (41 µm in height and 35 µm in diameter). In a different study, the impact of larger and more complex surface structures was investigated ex vivo and these were found to have a large impact on the adhesive properties of the microdevices [39].

When comparing the loaded and coated microcontainers to the empty reference microcontainers with and without pillars, it is important to consider the possible effect of the lid coating. Eudragit^®^ polymers have previously been found to possess mucoadhesive properties when applied on nanocapsules [48]. Thus, the coating itself could influence the adhesion of the microcontainers even if Eudragit^®^ L100 is expected to dissolve quickly at pH 7.4. However, all three types of cylindrical microcontainers appeared to result in similarly low mucoadhesion (Figure 5), which indicates that the shape is the most important factor for mucoadhesion in the present study.

In two euthanized rats dosed with cylindrical reference microcontainers, a remarkably smaller number of microcontainers were found to adhere after 30 min than for other rats dosed with reference cylinders. These findings indicate that the microcontainers interact differently with the colonic mucus in the presence of peristalsis, irrigation and water-resorption processes, which emphasizes the importance of evaluating mucoadhesion in situ as well as ex vivo.

#### 3.3.2. Absorption of Amoxicillin

**Figure 6:**
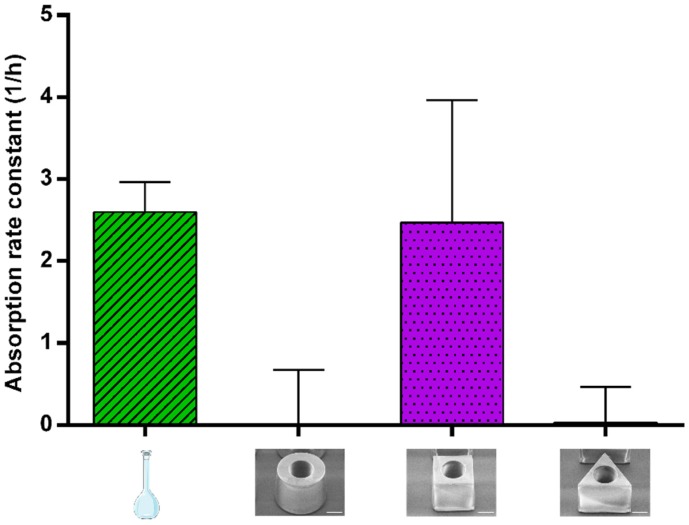
First-order absorption rate constants (k_a_) calculated from the closed-loop intestinal perfusion studies for Eudragit^®^ L100 coated microcontainers loaded with amoxicillin and for an amoxicillin control solution (mean ± SD, *n* = 6).

To address whether the mucus retention affected the absorption of amoxicillin from the microcontainers, blood and intestinal samples were collected. Based on the remaining concentrations in the intestinal lumen, the absorption rate constant (k_a_) was calculated for amoxicillin in solution and amoxicillin dosed via cylindrical, cubic and triangular microcontainers (Figure 6). The values for k_a_ are relevant in order to evaluate how mucoadhesion affects the absorption of amoxicillin.

For amoxicillin in cubic microcontainers, k_a_ was calculated to be 2.5 ± 0.6 h^−1^, which is not statistically different to the value obtained for the solution (2.6 ± 0.4 h^−1^) (Figure 6). For cylinders and triangular prisms, k_a_ of amoxicillin was calculated to be 0.0 ± 0.7 h^−1^ and 0.0 ± 0.5 h^−1^, respectively (Figure 6). In the case of the cubic microcontainers, absorption seems to be faster than the release, resulting in a positive k_a_. This could be related to the slower in vitro release observed from this shape (Figure 3). On the contrary, the concentrations of amoxicillin measured in the lumen after dosing with cylindrical and triangular microcontainers appeared to be constant during the whole experiment. This could indicate that the absorption and release occurred with the same rate, and, thus, the absorbed amount of amoxicillin was continuously replaced by the released amount.

After 30 min, a blood sample was collected to compare k_a_ to the amount of amoxicillin absorbed from the colon during the experiment. In plasma, amoxicillin was mainly detected after dosing in solution and cubic microcontainers (0.26 ± 0.03 and 0.08 ± 0.02 µg/mL, respectively). On the contrary, amoxicillin could only be detected in plasma from one of the six rats dosed with triangular microcontainers (resulting in 0.02 ± 0.02 µg/mL for the group on average). Systemic uptake of amoxicillin was not detected in any of the blood samples from the rats dosed with cylindrical microcontainers. The absorption of amoxicillin has previously been shown to vary in different regions of the GI tract with limited absorption in the colon [49]. These region-dependent differences in absorption are believed to be caused by decreased levels of the uptake transport responsible for the absorption of amoxicillin [49,50].

In summary, the control solution and the cubic microcontainers were the formulations with the highest k_a_, which also resulted in the highest concentration of amoxicillin in the plasma after 30 min. Amoxicillin dosed in the control solution had the obvious advantage that it was already in solution and available for absorption, whereas the amoxicillin powder inside the microcontainers needed more time to be released, solubilized and then absorbed. Based on the in vitro release profiles (Figure 3), only approximately 40% of the dose was expected to be released in the intestinal medium after 30 min, which could explain the observed difference in plasma concentrations. The different preconditions, but yet comparable performances, for the solution and the cubic microcontainers, suggested that the microcontainers must hold a different advantage, which might be related to the mucoadhesion. A high degree of mucoadhesion as observed for the cubic microcontainers would result in a high local concentration of amoxicillin facilitating the absorption.

Cylindrical microcontainers have previously been evaluated in an in situ closed loop perfusion model in the small intestine in order to investigate mucoadhesion and absorption of furosemide [16]. The microcontainers were found to adhere to the intestinal mucus and result in a higher absorption rate constant for furosemide when compared to a control solution [16]. The differences between this work and the present one can be attributed to the properties of the API and the intestinal section in which the absorption takes place.

## 4. Conclusions

In the present study, we investigated the influence of microdevice shape on colonic mucoadhesion and drug absorption by applying an in situ closed-loop intestinal perfusion technique. Cylindrical, triangular and cubic microcontainers were loaded with amoxicillin as a model drug and subsequently coated with Eudragit^®^ L100. The amoxicillin release was evaluated in vitro and the absorption of amoxicillin and adhesion of microcontainers was evaluated in a closed-loop intestinal perfusion model in anesthetized rats.

In vitro, a complete amoxicillin release was observed after 60 min from the three types of microcontainers. From the microscopy analysis of the colon sections after the in situ perfusion study, it was evident that a significantly higher percentage of cubic microcontainers than cylindrical microcontainers (33 ± 12% and 12 ± 7%, respectively) was detained in the mucus. Additionally, the absorption rate constants and the blood samples indicated that amoxicillin in cubic microcontainers was absorbed more readily (2.5 ± 0.6 h^−1^ and 0.08 ± 0.02 µg/mL, respectively) than when cylindrical microcontainers (0.0 ± 0.7 h^−1^ and no absorption detected) or triangular microcontainers (0.0 ± 0.5 h^−1^ and 0.02 ± 0.02 µg/mL) were dosed. This could be due to a higher degree of mucoadhesion for these particular microcontainers.

With the present study, we have demonstrated that the in situ closed-loop intestinal perfusion model is a promising tool to evaluate overall performance of microdevices in a confined region of a rat intestine. Based on the presented results, the use of more complex microcontainer shapes including more edges and corners, such as star shapes, should be investigated in the future.

## Figures and Tables

**Table 1:** Design parameters of the microcontainers evaluated in the present study. Data represents mean ± SD and *n* = 5 unless otherwise specified.

Shape	Topology Image	Inner Diameter (µm)	Outer Diameter/Side Length ^a^ (µm)	Inner Height (µm)	Outer Height (µm)	Surface Area (Normalized to Cylinder)
Cylindrical	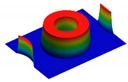	157.8 ± 2.2	304 ± 2.2	210.8 ± 3.1	247.8 ± 3.1	1.000 ^b^
Cubic	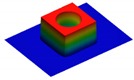	157.6 ± 2.2	248.0 ± 2.2	211.0 ± 4.6	245.0 ± 4.6	0.985
Triangular	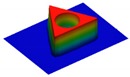	158.2 ± 2.2	342.0 ± 2.2	210.6 ± 4.2	245.4 ± 4.0	1.003
Cylindrical (reference) ^c^	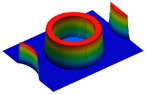	234.3 ± 2.2	324.7 ± 2.2	218.0 ± 3.0	252.0 ± 1.7	1.099
Cylindrical (reference) with pillars ^d,e^	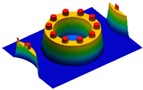	219.5 ± 2.2	323.7 ± 2.2	166.3 ± 1.5	202.8 ± 1.2	0.933

^a^ the diameter for all cylindrical microcontainers and the side length for the cubic and triangular microcontainers. ^b^ corresponding to 309243.3 µm^2^. ^c^ *n* = 3. ^d^ *n* = 6. ^e^ pillar dimensions: 41 µm high with a diameter of 35 ± 2.2 µm.
